# Comparison of lipophilic and size-exclusion membranes: creating sink conditions with cyclodextrin

**DOI:** 10.5599/admet.2859

**Published:** 2025-08-27

**Authors:** Petra Tőzsér, Szabina Kádár, Edina Szabó, Hajnalka Pataki, Péter Sóti, Péter Laczay, György T. Balogh, Bálint Sinkó, Enikő Borbás

**Affiliations:** 1 Department of Organic Chemistry and Technology, Faculty of Chemical Technology and Biotechnology, Budapest University of Technology and Economics, 3 Műegyetem Quay, H-1111, Budapest, Hungary; 2 Lavet Pharmaceutical Ltd., 6 Batthyány Street., H-2143, Kistarcsa, Hungary; 3 Department of Pharmaceutical Chemistry, Faculty of Pharmaceutical Sciences, Semmelweis University, 9 Hőgyes Endre Street., H-1092, Budapest, Hungary; 4 Center for Pharmacology and Drug Research & Development, Semmelweis University, 26 Üllői Street., H-1085, Budapest, Hungary; 5 Department of Chemical and Environmental Process Engineering, Faculty of Chemical Technology and Biotechnology, Budapest University of Technology and Economics, 3 Műegyetem Quay., H-1111, Budapest, Hungary; 6 Pion Inc., Billerica, 10 Cook Street, Massachusetts 01821, USA

**Keywords:** Unstirred water layer, flux, solubility, supersaturation ratio, carvedilol

## Abstract

**Background and purpose:**

The effective transport of an active pharmaceutical ingredient across various membrane systems is critical for enhancing its bioavailability, especially in formulations involving solubilizing agents. This study aims to investigate the permeability differences of carvedilol between lipophilic (organic solvent) and size-exclusion membranes in the presence of 2-hydroxypropyl-beta-cyclodextrin in just the acceptor compartment or both sides of the membrane using *in vitro* side-by-side diffusion cell assays.

**Experimental approach:**

Cyclodextrins (CDs) on the acceptor side significantly improved flux and permeability for the lipophilic membrane. In contrast, with size-exclusion membranes that allow the permeation of CDs and their complexes, the benefits of sink conditions were completely diminished. When the same amount of CD was introduced on both sides, the negative effect of CD on the donor side surpassed the positive sink effects on the acceptor side, resulting in reduced flux and permeability across all membrane types.

**Key results:**

A novel aspect of this work is the assessment of the applicability of a previously described general mathematical equation for sink conditions. Findings indicated that the supersaturation ratio between donor and acceptor compartments serves as the primary driving force of the membrane transport. For the lipophilic membrane, CDs on the acceptor side not only influenced the driving force of the transport by enhancing the solubility of carvedilol in the acceptor compartment but also altered the proportionality coefficient, hence modifying the apparent thickness of the unstirred water layer. The impact was not observed with size-exclusion membranes. The applicability of the mathematical model was additionally evaluated for CD placed on both sides of the membrane.

**Conclusion:**

The model effectively describes the impact of CD placed on the donor side when the solid membrane permits only the drug’s permeation, as in the case of a lipophilic membrane, where the solubilizing additive cannot pass through. It is also applicable when the solubilizing additive permeates slowly and has minimal influence on transport, such as with a size-exclusion membrane with a 1 kDa molecular weight cut-off. The model remains suitable if the additive is small enough in hydrodynamic size to permeate the membrane, but no concentration gradient exists to drive its transport, for example, with a 6 kDa size-exclusion membrane containing the same CD concentration on both sides of the membrane.

## Introduction

This study builds upon our previous research [1], which investigated the flux differences between lipophilic and size-exclusion membranes in the presence of 2-hydroxypropyl-beta-cyclodextrin (HP-β-CD), focusing on the impact of stirring and the addition of solubilizing agents in the donor compartment. However, it primarily examined cases where HP-β-CD was added to the donor side, leaving open the question of how permeability and the driving force of membrane transport change when HP-β-CD is placed in the acceptor compartment or in both compartments simultaneously. HP-β-CD is frequently used not only as a formulation additive but also in the acceptor medium to provide sink conditions [2-6] in dissolution-permeation testing. Even in this case, HP-β-CD functions by forming inclusion complexes with the drug, thus increasing the aqueous solubility and preserving a consistent concentration gradient between the donor and acceptor sides.

The concept of sink condition is fundamental in the field of drug dissolution and permeation studies. Sink conditions denote a state in which the concentration of a dissolved drug in the acceptor compartment remains significantly lower than the saturation solubility, hence preserving a constant driving force for drug diffusion and ensuring that the absorption rate is unaffected by the concentration gradient [7,8]. This concept originates from Walter Nernst [9], who described the impact of concentration gradients on the diffusion rate in his work on diffusion laws. Although Nernst did not explicitly employ the phrase sink condition, his equations established an initial basis for comprehending how maintaining low concentrations in the receiving phase aids continuous diffusion. The term and its specific application to drug dissolution became more widely recognized through the work of Higuchi in the 1960s. Higuchi highlighted the importance of maintaining sink conditions to ensure accurate measurement of drug dissolution rates, emphasizing that without sink conditions, the release rate of drugs could be significantly underestimated. His research was pivotal in adapting the concept for use in pharmaceutical sciences, providing a clear methodology for studying the release of drugs from formulations [10-12]. Alex Avdeef expanded the comprehension of sink conditions within permeability studies. In his 2003 book, Avdeef [13] revealed the essential function of sink conditions in in vitro permeability assays, such as Caco-2 and PAMPA models. He argued that maintaining sink conditions is crucial for accurately representing physiological conditions, as it prevents the dissolution rate from being limited by the accumulation of drugs in the receiver solution. Avdeef also noticed that in the absence of sink conditions, the observed permeability may underrepresent a drug's real absorption capacity, particularly for poorly soluble molecules. Solubilizing agents like sodium lauryl sulphate (SLS) and complexing agents like HP-β-CD are often used in acceptor media to create sink conditions [3,13-18]. While micelle-forming agents generally enhance the solubility of poorly water-soluble drugs, for complexing agents to be effective solubilizers, they need to have molecular interaction, which depends on the size of the molecule; therefore, their effectiveness is highly selective. Carvedilol (CAR), a non-selective β-receptor blocker, was chosen as the model active pharmaceutical ingredient (API), while as the model solubilizing additive with known size and attributes, HP-β-CD was chosen, which shows the highest complexation efficiency (CE) with CAR among the CDs [19-21].

Lipophilic membranes, typically phospholipid-coated filters [22-30], are widely used to mimic passive transcellular diffusion across the epithelial layer of the gastrointestinal (GI) tract. These membranes simulate the lipid bilayer of cell membranes, requiring drugs to partition into a lipophilic phase before diffusing into the acceptor side. Conversely, size-exclusion membranes [31-41] function based on molecular size, allowing only small molecules to diffuse through their water-filled pores. Since these membranes cannot differentiate between ionized and neutral molecules, due to the absence of a lipophilic domain, the permeability shows no pH dependence [1]. This fundamental difference in transport mechanisms raises the question of whether size-exclusion membranes can serve as possible alternatives to lipophilic membranes for permeability assessments, particularly in cases involving solubilizing agents.

Transport across both membrane types can be membrane-limited but also can be influenced by the unstirred water layer (UWL), which creates an additional diffusional barrier at the membrane interface. In lipophilic membranes, drug molecules must overcome the UWL before partitioning into the lipophilic phase, while in size-exclusion membranes, the transport occurs entirely in an aqueous environment. However, these two aqueous environments may differ significantly because of the drug's molecular hydration and possible interaction of the drug and filter material. Consequently, for size-exclusion membranes, both the UWL and the membrane permeation means aqueous diffusion and no diffusion through a lipophilic phase is involved. When using the same apparatus (side-by-side diffusion cell), it can be assumed that the UWL permeabilities and the thickness of the UWL are the same for lipophilic and size-exclusion membranes when the same donor, acceptor media and stirring speed are applied [1].

In the previous publication, it was found that regardless of the limitation of the transport, the supersaturation ratio (SSR, defined as the ratio of the drug concentration present in solution to its thermodynamic solubility measured in exactly the same media) is the driving force of membrane transport. This statement was supported with a concentration-based mathematical description and experimental data for pure API in both compartments of the side-by-side diffusion cell and for complexing agent placed in the donor compartment with lipophilic membrane, where only the drug molecules could go through the membrane, and also with 1 kDa molecular weight cut-off (MWCO) size-exclusion membrane, where the permeation of the complexing agent is quite slow, so it does not affect the transport of the API substantially. In the case the complexing agent (HP-β-CD) is transported through the membrane in a significant amount (using a size-exclusion membrane with 6 kDa MWCO), which causes a significant deviation from the general mathematical model. In conclusion, hence the fundamental difference in transport mechanisms between lipophilic and size-exclusion membranes, a 1 kDa MWCO membrane could serve as a possible alternative to lipophilic membranes for permeability assessments in cases involving HP-β-CD in the donor compartment [1].

While the effect of HP-β-CD in the donor compartment was previously published, this study aims to investigate the differences in supersaturation ratio and permeability of the model API, CAR, between lipophilic (n-dodecane) and size-exclusion membranes, with HP-β-CD present in either the acceptor compartment or both sides of the membrane, using in vitro side-by-side diffusion cell assays. The additional objective of this research is to assess the applicability of a previously described general mathematical equation and determine the driving force of membrane transport under sink conditions.

## Experimental

### Materials

**Figure 1.**
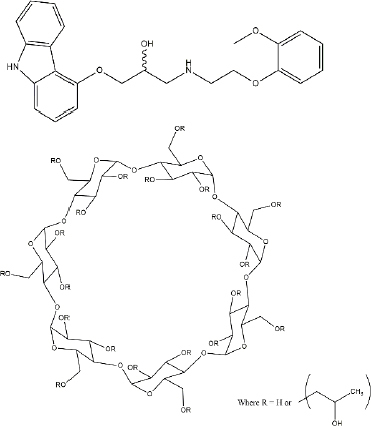
The structural formula of CAR and HP-β-CD

Carvedilol (CAR) [43], a poorly water-soluble but well-permeable (BCS Class II), monovalent weak base with a p*K*_a_ of 7.97 (25 °C, 0.15 M ionic strength) [42], (406.482 g mol^-1^, structure shown in Figure 1.), was obtained from Sigma-Aldrich Co. LLC. (St. Louis, MO, USA). HP-β-CD, known as a solubilizing agent, is a doughnut-shaped molecule composed of seven alpha-D-glucopyranose units[44], including an internal cavity (DS: 4.34, structure shown in Figure 1), was obtained from Roquette Fréres (Lestrem, France).

Prisma^HT^ buffer and polyvinylidene fluoride (PVDF) sheet with 0.45 μm pore size were purchased from Pion Inc. (Billerica, MA, USA), *n*-dodecane from Sigma-Aldrich (St. Louis, Missouri, USA) and methanol from Thomasker (Budapest, Hungary). 1 kDa and 6 to 8 kDa regenerated cellulose size-exclusion membranes were purchased from Repligen (Boston, MA, USA).

### *In vitro* side-by-side diffusion cell assays

*In vitro* side-by-side diffusion cell assays were performed with μFLUX (Pion Inc., Billerica MA, USA) apparatus. It is composed of a donor and an acceptor chamber (20 mL volumes) separated by an artificial membrane selected to be a size-exclusion regenerated cellulose membrane (MWCO 6 kDa and 1 kDa, 1.54 cm^2^,) pre-soaked in distilled water overnight prior to measurement, and a lipophilic membrane (0.45 μm pore size PVDF sheet, 1.54 cm^2^) impregnated with 25 μL *n*-dodecane. The measurements were performed in four different arrangements for each membrane type, which are listed in Table 1.

**Table 1.** *In vitro* side-by-side diffusion cell assay measurement settings

Settings No.	Donor side	Acceptor side
1.	18 mL pH 10 Prisma buffer	18 mL pH 10 Prisma buffer
2.	18 mL pH 10 Prisma buffer + 15 mg mL^-1^ HP-β-CD	18 mL pH 10 Prisma buffer
3.	18 mL pH 10 Prisma buffer	18 mL pH 10 Prisma buffer + 15 mg mL^-1^ HP-β-CD
4.	18 mL pH 10 Prisma buffer + 15 mg mL^-1^ HP-β-CD	18 mL pH 10 Prisma buffer + 15 mg mL^-1^ HP-β-CD

The appropriate amount of 3 mg mL^-1^ methanol stock solution of the drug was pipetted to the donor side. Both cells were thermostated and stirred at 250 rpm based on the results of our previous study, which showed that stirring is essential to investigate the effect of excipients [1]. Drug concentrations at the donor and acceptor sides were monitored in real-time using UV probes with the Rainbow (Pion Inc., Billerica, MA, USA) apparatus. A second derivative method between 292 and 300 nm was used to evaluate the spectra, and the flux (*J*) and apparent permeability (*P*_app_ / cm s^-1^) were calculated [13] according to Equation 1:



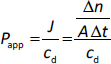

(1)


where apparent permeability (*P*_app_) is the quotient of flux (*J* / mol s^-1^ cm^2^) and donor-side concentration (*c*_d_ / mol cm^-3^), flux (*J*) of a drug across the membrane is defined as the molar amount of substance diffused from the donor to the acceptor compartment (*n* / mol) crossing a unit area (*A* / cm^2^) perpendicular to its flow per unit time (*t* / s).

## Results and discussion

This work investigates the effect of sink condition across three distinct membrane types (PVDF filter impregnated with *n*-dodecane and two size-exclusion membranes MWCO = 1 and 6 kDa). For that, solubility measurements were carried out to see if HP-β-CD can truly create a sink condition for the model drug CAR (see detailed results in the previous publication [1]). The concentration of the solubilizing additive was chosen based on a known HP-β-CD containing marketed solution [45] and the recommendation to take the solution with a glass of water. The thermodynamic solubility results with the chosen 15 mg mL^-1^ HP-β-CD concentration showed three times higher value than the solubility of the pure API in plain pH 10 buffer [1].

The fundamental difference between the membrane types is that in the case of the lipophilic membrane, only the drug can permeate the membrane [1], while the solubilizing agent and the drug-CD complex cannot. In contrast, when using size-exclusion membranes, both the drug and the HP-β-CD, as well as their complex, can permeate. The transport was found 1.4 times faster in the case of the 6 kDa than in the case of the 1 kDa MWCO membrane [1].

### *In vitro* side-by-side diffusion cell assay

Considering the apparent permeability-pH profile of CAR [1], pH 10 media was chosen for the *in vitro* side-by-side diffusion cell assays to create UWL-limited transport conditions when a lipophilic membrane is used in the absence of HP-β-CD. The previous study also revealed that the effect of complexing agents can be better characterized at stirred conditions compared to a non-stirred environment; therefore, 250 rpm stirring was chosen for the following diffusion cell assays [1].

**Figure 2.**
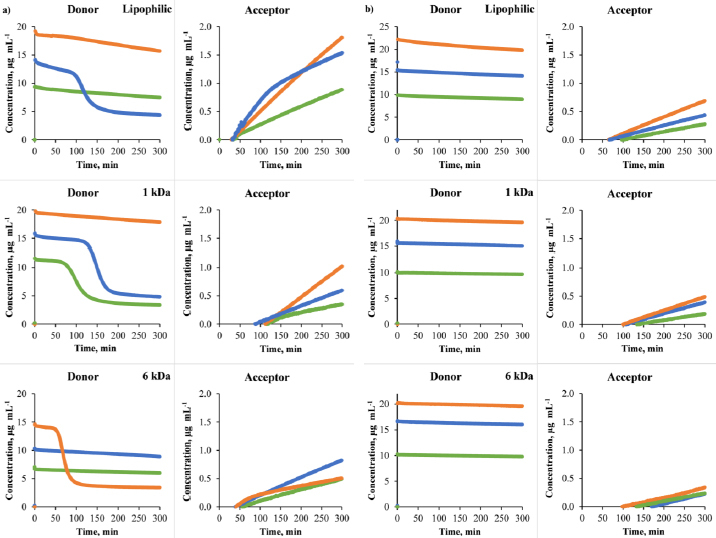
Examples of donor and acceptor concentration – time profiles of CAR in pH 10 Prisma buffer: a) HP-β-CD present on the acceptor side of the membrane b) HP-β-CD present on both sides of the membrane for the lipophilic membrane, 1 kDa size-exclusion membrane, and 6 kDa size-exclusion membrane data coming from the same diffusion cell assay is marked with the same colours)

Figure 2 shows examples of concentration-time profiles to present the measurement results for three different targeted donor concentrations in the donor and acceptor compartments for lipophilic membrane, 1 and 6 kDa membranes. Donor concentration curves exhibit a linear decline as the API is transported to the acceptor side. Thermodynamic solubility of CAR Form I is 2.15 μg mL^-1^ in plain pH 10 buffer, 6.69 μg mL^-1^ in HP-β-CD containing buffer [1]. The API was introduced to the donor side from a methanol stock solution and given that the concentration of the API varies from 0 to 25 μg mL^-1^, in most experiments, the donor concentration exceeded the thermodynamic solubility; therefore, supersaturated solutions of CAR were created in the donor compartment. In certain cases (Figure 2a), the API precipitated from these supersaturated solutions (Figure 2), resulting in a rapid decline in both the donor concentration and the slope of the acceptor concentration-time curve. The curves on the acceptor side predominantly exhibit linearity, with a substantial reduction in the slope observable solely during precipitation on the donor side. Flux was calculated from the slopes of the lines fitted to their initial linear phases.

**Figure 3.**
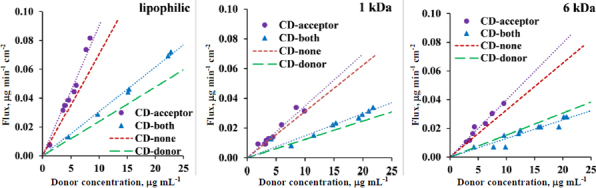
Flux – donor concentration curves in pH 10 buffer with 250 rpm stirring rate for lipophilic membrane, 1 and 6 kDa size-exclusion membranes. No HP-β-CD present on either side of the membrane with red dotted line called ‘CD-none’, addition of HP-β-CD to acceptor side with purple dots called ‘CD-acceptor’, addition of HP-β-CD to donor side with green dashed line called ‘CD-donor’ and addition of HP-β-CD to both sides with blue triangles called ‘CD-both’

**Figure 4.**
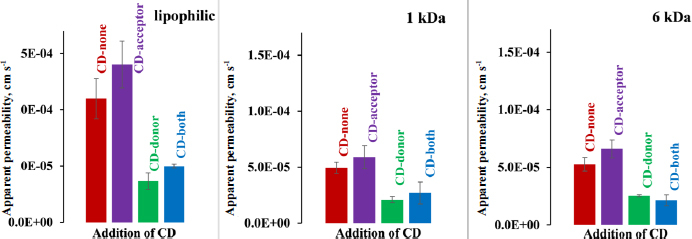
Apparent permeability values in a pH 10 buffer with a 250 rpm stirring rate in the case of lipophilic membrane, 1 and 6 kDa size-exclusion membranes. No HP-β-CD present on either side of the membrane with **red**, addition of HP-β-CD to acceptor side with **purple**, addition of HP-β-CD to donor side with **green** and addition of HP-β-CD to both sides with **blue**

The flux values obtained from *in vitro* side-by-side diffusion cell assays measurements were plotted as a function of CAR concentration in the donor compartment (Figure 3). Figure 3 shows that the addition of CD to the acceptor solution results in a modest increase in the slope of the flux curve when comparing the ‘CD-acceptor’ curve to the ‘CD-none’ curve, and the ‘CD-both’ curve to the ‘CD-donor’ curve across all three membrane types, attributable to sink condition. The slope of the flux-donor concentration line is directly proportional to the permeability value, which was calculated and plotted in Figure 4. The sink effect is significant using a lipophilic membrane (see Table 2 for the homogeneity of slopes test *p*-value for ‘CD-none’ and ‘CD-acceptor’ *p* < 0.05). However, for both size-exclusion membranes, the sink effect is negligible, most probably due to CD’s ability to pass through the membrane from the acceptor side to the donor side. A similar situation arises when CD in the donor compartment is compared with the scenario where both chambers contain CD (Table 2, *p*-value for ‘CD-donor’ and ‘CD-both’): in case of the lipophilic membrane, the sink effect is significant; however, it is negligible for size-exclusion membranes.

**Table 2.** Homogeneity of slopes test results for flux-donor concentration curves in pH 10 buffer with 250 rpm stirring rate for lipophilic membrane, 1 and 6 kDa size-exclusion membranes, significance: *p* < 0.05 is highlighted in red

	*p*-value for CD-none and CD-acceptor	*p*-value for CD-donor and CD-both
Lipophilic	0.001748	0.000562
1 kDa	0.689878	0.594536
6 kDa	0.176109	0.127748

Notably, despite the same amount of CD applied to both the donor and acceptor side, the CD on the donor side exerts a significantly greater negative effect on flux and therefore permeability (Figures 3 and 4) than the positive effect of CD used to create sink conditions in all 3 membrane types. Therefore, in the scenario where both sides contain CD, the transport of the API is much slower than in the case where no CD is added to either side of the membrane (see Figures 3 and 4, CD-none case), regardless of the type of membrane. For a detailed description of the effect of CD used in the donor compartment, see the previous publication [1].

From the apparent permeability values depicted in Figure 4 and the results of pH-permeability profiling in previous publication [1], the *P*_UWL_, *P*_m_ values were calculated (Table 3) assuming that: a) the UWL exist symmetrically on both sides of the membrane, b) the thickness of the UWL is the same for different membranes when using the same side-by-side diffusion cell with the same experimental conditions (pH of media, HP-β-CD in media, stirring speed).

**Table 3.** Summary table of *P*_app_, *P*_UWL_, *P*_m_ values from the *in vitro* side-by-side diffusion cell assay, 250 rpm

	Lipophilic membrane				1 kDa size-exclusion membrane				6 kDa size-exclusion membrane			
	CD-none	CD-donor	CD-acceptor	CD-both	CD-none	CD-donor	CD-acceptor	CD-both	CD-none	CD-donor	CD-acceptor	CD-both
*P*_app_ / 10^-5^ cm s^-1^	10.99	3.66	14.02	5.00	4.94	2.09	5.83	2.68	5.26	2.54	6.60	2.15
*P*_e_ / 10^-5^ cm s^-1^	n.a.	7.03	14.54	8.33	n.a.	n.a.	n.a.	n.a.	n.a.	n.a.	n.a.	n.a.
*P*_m_ / 10^-5^ cm s^-1^	28.73	9.23*	28.73*	9.23*	6.84*	2.25*	7.27*	2.77*	7.47*	2.78*	8.51*	2.21*
*P*_UWL_ / 10^-5^ cm s^-1^	17.80*	29.44	29.44*	85.14	17.80	29.44	29.44	85.14	17.80	29.44	29.44	85.14
*P*_UWL donor_ / 10^-5^ cm s^-1^	35.60	170.29	35.60	170.29	35.60	170.29	35.60	170.29	35.60	170.29	35.60	170.29
*P*_UWL acceptor_ / 10^-5^ cm s^-1^	35.60	35.60	170.29	170.29	35.60	35.60	170.29	170.29	35.60	35.60	170.29	170.29
*D*_UWL donor_ / 10^-5^ cm s^-1^	0.65	0.44	0.65	0.44	0.65	0.44	0.65	0.44	0.65	0.44	0.65	0.44
*D*_UWL acceptor_ / 10^-5^ cm s^-1^	0.65	0.65	0.44	0.44	0.65	0.65	0.44	0.44	0.65	0.65	0.44	0.44
*h*_UWL donor /_ μm	183	26	183	26	183	26	183	26	183	26	183	26
*h*_UWL acceptor_ / μm	183	183	26	26	183	183	26	26	183	183	26	26

*if *P*_UWL_ < *P*_m_ then the system tends towards UWL limitation, these cases have blue background; if *P*_UWL_ > *P*_m_ then the system tends towards membrane limitation, these cases have green background; if there was no significant difference found between the P_m_ and P_UWL_ values then beige background was used.

From the results in Table 3 it can be concluded that, when only plain pH 10 media is used on both sides of the lipophilic membrane, then the membrane transport is UWL limited. When HP-β-CD is placed on the acceptor side, then the diffusion through the membrane and the UWL have similar speed, and when both compartments contain the complexing agent, then the transport becomes membrane-limited. In case of size-exclusion membranes, regardless of the MWCO, the limiting step of transport is the diffusion through the water-filled membrane. This holds for all experimental scenarios: when no HP-β-CD is present on either side (Table 3 CD-none), when HP-β-CD is present on the donor side (Table 3 CD-donor), when HP-β-CD is present on the acceptor side (Table 3 CD-acceptor), and when HP-β-CD is present on both sides of the membrane (Table 3 CD-both).

### Discussion of the driving force of membrane transport in case of sink condition

In this section, the aim was to assess the applicability of a previously described general mathematical Equation (1) under sink conditions. This equation was formulated to describe the transport across the UWL-membrane-UWL system in a general way (Equation (2)), without defining the specific type of membrane located between the two UWL layers or specifying the limiting step of transport. Experimental results confirmed the applicability of the model when the membrane is selectively permeable for the drug (lipophilic membrane) or when the permeation of the solubilizing additive is negligibly slow (1 kDa MWCO ) [1]. The mathematical model is now applied in cases where the solubilizing additive is placed on the acceptor side to create sink conditions, or where the CD is added to both sides of the lipophilic and size-exclusion membranes.



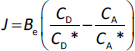

(2)


where *J* is the flux, *B*_e_ is the effective coefficient of proportionality, *C*_D_ and *C*_A_ are the concentrations, and *C*_D_* and *C*_A_* are the solubility values in the donor and acceptor media [1].

Equation (2) indicates that the driving force of membrane transport is a difference in the supersaturation ratio (SSR) between the donor and acceptor compartments. This difference is directly proportional to the flux and the coefficient of proportionality, *B*_e_.

Equation (3) describes the reciprocal relationship between the coefficients of proportionality [1]:



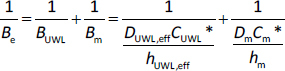

(3)


where *B*_UWL_is the *B* factor for the UWL and *B*_m_ is the *B* factor for the membrane, *D*_UWL,eff_ is the effective diffusion coefficient through the UWL and *h*_UWL,eff_ is the effective thickness of the UWL in the presence of HP-β-CD and *C*_UWL_ is the concentration of the API in the UWL, and * is the property at saturation, *D*_m_ is the diffusion coefficient through the membrane, *C*_m_ is the concentration of the drug in the membrane and * is the property at saturation, *h*_m_ is the membrane thickness.

**Figure 5.**
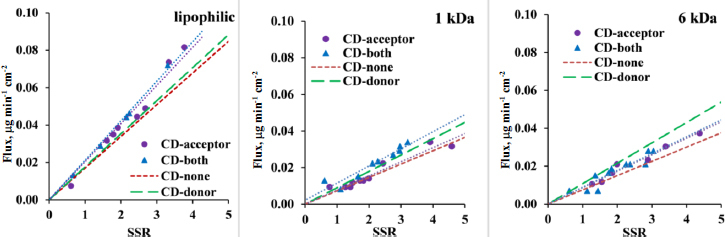
Flux – SSR curves in pH 10 buffer with 250 rpm stirring rate for lipophilic membrane, 1 and 6 kDa size-exclusion membranes. No HP-β-CD present on either side of the membrane with **red dotted line** called ‘**CD-none**’, addition of HP-β-CD to acceptor side with **purple dots** called ‘**CD-acceptor**’, addition of HP-β-CD to donor side with **green dashed line** called ‘**CD-donor**’ and addition of HP-β-CD to both sides with **blue triangles** called ‘**CD-both**’

Figure 5 shows the flux plotted as a function of the difference in SSR. The slopes of the lines equal the coefficient of proportionality. Homogeneity of slopes tests were carried out to see if the line of the ‘CD-none’ buffer on both sides (red) and the HP-β-CD containing buffer on the acceptor side (purple) differ significantly (significance: *p* < 0.05). The statistical analysis indicates that parallel lines are obtained by size-exclusion membranes, regardless of the MWCO size of the membrane (refer to *p*-values for CD-none and CD-acceptor in Table 4), while a significant difference is seen in the case of the lipophilic membrane. In a similar situation, where CD in the donor compartment is compared with the scenario in which both sides contain CD (Table 4, *p*-values for donor and both): in the case of the lipophilic membrane, the deviation from the parallel lines is significant, but for the size-exclusion membranes, it is not. In conclusion, for the lipophilic membrane, where the sink effect significantly influenced permeability (Figure 4), a significantly greater coefficient of proportionality (*B*_e_) (slope of the purple CD-acceptor curve on Figure 5) is observed compared to the ‘CD-none’ buffer (slope of the red curve on Figure 5).

**Table 4.** Homogeneity of slopes test results for flux-SSR curves in pH 10 buffer with 250 rpm stirring rate for lipophilic membrane, 1 kD and 6 kDa size-exclusion membranes

	*p*-value for CD-none and CD-acceptor	*p*-value for CD-donor and CD-both	*p*-value for CD-acceptor and CD-both
Lipophilic	0.000787	0.001689	0.819284
1 kDa	0.923485	0.853885	0.155029
6 kDa	0.133241	0.129204	0.735795

This can be explained by Equation (3): specifically, when a solubilizing agent is added to the acceptor side, the solubility in the acceptor compartment increases *C*_A_*, which is factored into the calculation of the difference in supersaturation ratio (*x*-axis on Figure 5). Also, the solubility of API in the UWL *C*_UWL_* is elevated (3 times higher) compared to the ‘CD-none’ buffer, due to the presence of the CDs in the aqueous boundary layer of the acceptor side. When the API is diffusing through the UWL as a CD complex, the molecular weight is increased, resulting in slower diffusion and a reduction in *P*_UWL_ value. However, the CD complex is not only bigger in size, but also much more hydrophilic than the API; therefore, one of the main assumptions of the calculations [1,46] is that only the free drug encounters a significant unstirred water layer thickness, but the unstirred water layer thickness is negligible for the CAR/HP-β-CD complex. This assumption leads to higher *P*_UWL_ values in the presence of CD and also causes the apparent *D*_UWL_ value to decrease (Table 3). These cause an increase in the numerator of the *B*_UWL_ value (Equation (3)). In literature, the addition of CD has been previously noted to cause a reduction of the apparent UWL thickness, which may also be interpreted as an elevation of the proportionality coefficient (B_UWL_) [36]. These effects can be seen in Figure 5: the purple curve becomes steeper than the red curve belonging to the CD-none buffer.

In a similar situation, when CD is present in the donor compartment versus a scenario where both sides contain CD, the same explanation can be applied using Equation (3).

Lipophilic and size-exclusion membranes are mechanistically different: in the case of lipophilic membranes, only the drug can permeate the membrane, while in the case of size-exclusion membranes, also the CD and the drug-CD complex are able to go through. A prior study [1] demonstrated that the transport of the HP-β-CD across a 1kDa MWCO membrane is quite slow, becoming detectable on the opposite side after 4 hours. In contrast, the transport across a 6 kDa MWCO membrane was found to be substantially faster, reaching the detection threshold in merely 2 hours. When the CD is placed on the acceptor side to create a sink condition and a size-exclusion membrane is used, the CD can cross the membrane and appear on the donor side, while the API moves in the opposite direction. Figure 5, along with the results of Table 4, indicates that there is no significant difference in the slopes of the CD-none buffer (red) and CD placed on the acceptor side (purple). Similarly, the CD on the donor side (green) and the CD on both sides (blue) also exhibit perfectly parallel lines. These results show that when the solid membrane dividing the two aqueous boundary layers becomes permeable to the CD, the sink effect shown with the lipophilic membrane reduces, and no significant deviation is evident in the proportionality coefficients.

In drug formulation testing, the most realistic scenario occurs when the formulation releases solubilizing agents in the donor compartment, while surfactants or CDs are employed in the acceptor compartment to establish sink conditions. Therefore, the effect of placing a CD on the donor side while already having a CD on the acceptor side was evaluated in Figure 5. This means the comparison of the cases labelled ‘CD-acceptor’ in purple and ‘CD-both’ in blue in Figure 5. According to the homogeneity of slopes tests in the case of all three membrane types, the purple and blue lines are found to be parallel, which means that the effect of CD placed in the donor compartment can be well described by Equation (2), regardless of the type of solid membrane. A similar scenario was previously described [1] in which the applicability of Equation (2) was evaluated with CD placed in the donor compartment and ‘CD-none’ buffer utilized as acceptor media. It was determined that Equation (2) effectively represented the results for the lipophilic and size-exclusion membrane with 1 kDa MWCO; however, for the size-exclusion membrane with 6 kDa MWCO, the transport of the CD through the membrane resulted in a significant deviation in the proportionality coefficient. However, when the acceptor side already contains an equivalent concentration of CD as the donor side, a concentration gradient does not exist to drive the transport of the solubilizing additive, even though the membrane is permeable to CDs. Consequently, no significant difference is observed in the coefficient of proportionalities, even with the size-exclusion membrane with 6 kDa MWCO, when comparing the ‘CD-acceptor’ setup with both instances (third column of Table 4).

## Conclusions

In this study, the complex interplay between solubility enhancement and membrane permeability in side-by-side diffusion cell assays was explored, focusing on the effects of HP-β-CD, creating sink conditions. For the lipophilic membrane, CDs on the acceptor side significantly enhanced flux and permeability. Conversely, with size-exclusion membranes that allow the permeation of CDs and their complexes, the benefits of sink conditions were completely diminished. The introduction of CDs on both sides resulted in the negative impacts on the donor side surpassing the beneficial sink effects on the acceptor side, hence reducing flux and permeability for all membrane types.

A novel aspect of this work is the assessment of the applicability of a previously described general mathematical equation for sink conditions. This equation was developed to describe the transport through the UWL-membrane-UWL system in a general way, without specifying what type of membrane is between the two UWL layers. This model was used to describe the effect of CD in the donor compartment on the API transport for lipophilic and size-exclusion membranes with MWCOs 1 and 6 kDa. Experimental results confirmed the applicability of the model when the membrane is selectively permeable for the drug (lipophilic membrane) or when the permeation of the solubilizing additive is negligibly slow (1 kDa MWCO ) [1]. In this paper, the applicability of this model was further assessed in sink conditions. For the lipophilic membrane, where the sink effect had a significant impact on the permeability, a significantly higher proportionality coefficient was observed with CD placed on the acceptor side compared to the ‘CD-none’ buffer. This phenomenon might be explained by the fact that CD not only influences the solubility of the API on the acceptor side, but also the hydrophilicity of the drug-CD complex is much higher than that of the pure API’s; therefore, the thickness of the UWL becomes negligible for the complex. This leads to reduced apparent UWL thickness (*h*) and increased *P*_UWL_ values (Table 3) [46]. In contrast, with size-exclusion membranes, where the CDs and their complexes can permeate the membrane, the effect of the sink condition was negated, resulting in no significant difference in the coefficient of proportionality.

In formulation development, the typical scenario involves the formulation releasing solubilizing additives in the donor compartment while surfactants or CDs are used in the acceptor compartment to create a sink condition. Therefore, the effect of placing the same amount of CD on the donor side as on the acceptor side was also evaluated. The effect of the CD placed in the donor compartment can be accurately described by the general mathematical equation, regardless of the solid membrane type. These results were compared with previously published data, concluding that this universal equation effectively describes the impact of CD placed on the donor side. This occurs when the solid membrane permits only the drug’s permeation, as in a lipophilic membrane, where the solubilizing additive is unable to go through due to its size or lipophilicity. It also applies when the permeation of the solubilizing additive is sufficiently slow, thus not significantly affecting the transport, as observed with a size-exclusion membrane with 1kDa MWCO. Furthermore, the model remains suitable if the additive is small enough in hydrodynamic size to permeate the membrane yet lacks a concentration gradient to drive the transport of the solubilizing agent, such as with 6 kDa MWCO size-exclusion membrane with the same CD concentrations on both sides of the membrane. ‘

In conclusion, this study elucidates a deeper understanding of the mechanistic effects of CDs in side-by-side diffusion cell assays, providing a valuable framework for optimization of drug formulations.
